# Dual-target drugs against *Leishmania donovani* for potential novel therapeutics

**DOI:** 10.1038/s41598-023-45448-x

**Published:** 2023-10-26

**Authors:** Kushal Bora, Manash Sarma, Shankar Prasad Kanaujia, Vikash Kumar Dubey

**Affiliations:** 1 School of Biochemical Engineering, Indian Institute of Technology (BHU), Varanasi, Uttar Pradesh 221005 India; 2 Department of Biosciences and Bioengineering, Indian Institute of Technology Guwahati, Guwahati, Assam 781039 India

**Keywords:** Computational biology and bioinformatics, Drug discovery

## Abstract

Antioxidant defense mechanisms are important for a parasite to overcome oxidative stress and survive within host macrophage cells. Mitochondrial iron superoxide dismutase A (FeSODA) and trypanothione reductase (TR) are critical enzymes in the antioxidant defense mechanism of *Leishmania donovani*. FeSODA is responsible for neutralizing reactive oxygen species in mitochondria, while TR is responsible for reducing trypanothione, the molecules that help the parasite fight oxidative stress in Leishmania. In this study, we used multitarget ligands to inhibit both the FeSODA and TR enzymes. We combined structure-based drug design using virtual screening approach to find inhibitors against both the targets. The ZINC15 database of biogenic compounds was utilized to extract drugs-like molecules against leishmaniasis. The compounds were screened by standard precision (SP) and extra precision (XP) docking methods. Two compounds, ZINC000008876351 and ZINC000253403245, were selected based on molecular docking based on the binding affinity for both the targets. The screened molecules ZINC000008876351 and ZINC000253403245 showed strong hydrogen bonding with the target proteins according to the Molecular mechanics with generalised Born and surface area solvation (MM-GBSA) techniques. These two compounds were also experimentally investigated on promastigotes stage of *L. donovani*. Under in vitro condition, the compounds show inhibitory effects on *L. donovani* promastigotes with IC_50_ values of 24.82 ± 0.61 µM for ZINC000008876351 and 7.52 ± 0.17 µM for ZINC000253403245. Thus, the screened compounds seem to have good potential as therapeutic candidates for leishmaniasis.

## Introduction

Infectious diseases rank at the third leading cause of death worldwide, encompassing numerous neglected diseases. Leishmaniasis, a parasitic disease, is one such disease which is prevalent in subtropical and tropical regions of Asia, Africa, Americas, and Europe. World Health Organization (WHO) has classified it as a neglected tropical disease^1^. The global population harbors an estimated 10–12 million individuals infected with leishmaniasis, with an annual incidence of 0.9–1.6 million cases leading to 20,000–50,000 deaths^2^. Leishmaniasis manifests in three clinical forms, viz. cutaneous, subcutaneous, and visceral^2^. Visceral leishmaniasis (VL) is caused by *Leishmania donovani*, a single-cell protozoan. VL is the most severe form of the disease. In India, Bangladesh, Nepal, Ethiopia, Sudan, and Brazil worlds, 90% of VL cases are reported to be caused by *Leishmania donovani* and *Leishmania* related species. Over 83 countries, more than 30,000 new VL cases are reported annually^3^. VL is characterized by persistent fever, hepatomegaly, splenomegaly, pancytopenia, anemia, and weight loss. The transmission of leishmaniasis occurs through the bite of infected sand flies, which transmit the obligate digenic protozoan parasite known as Leishmania. The protozoan undergoes two developmental stages, one within the insect vector and the other as an amastigote within the mammalian host. Inside the host's phagolysosomes of macrophages, the parasite replicates and transforms into an intracellular amastigote^4,5^.

The existing treatment for leishmaniasis has side effects, incurs high costs, lacks durability and resistance towards certain drugs. Drugs currently used include miltefosine, amphotericin B, liposomal amphotericin B, pentamidine, paromomycin, and pentavalent antimony derivatives (e.g. sodium stibogluconate, N-methylglucamine antimonate). The parasite has gained resistance towards pentavalent antimony and miltefosine, while liposomal amphotericin and paromomycin are expensive. Some drugs also have severe side effects, such as pentavalent antimony and miltefosine^6^. Given the limitations of conventional chemotherapeutic drugs and the absence of a leishmaniasis vaccine, there is a pressing need to develop new strategies and identify novel drug molecules for the treatment of leishmaniasis. The complexity arises from the fact that leishmaniasis lacks a single biological target. Often, the parasite develops resistance to drugs targeting a single biological target and employs alternative pathways to evade the drug's effects. Therefore, the development of anti-leishmaniasis drugs with multiple targets holds promise as they are less susceptible to targeting mechanisms. In recent decades, the dominant paradigm in drug discovery has been the “one target, one drug” approach, assuming that modulating a single disease-related biological target can effectively control disease symptoms or progression^7–10^. However, approximately twenty years ago, a group of pioneering researchers proposed the efficient utilization of small organic molecules with multitarget profiles. Their work demonstrated that specially designed compounds can possess a mechanism of action that targets multiple biological sites^11^. Since then, the field of multitarget drug discovery has rapidly evolved, introducing new paradigms that have the potential to overcome the limitations associated with classical single-target strategies.

**Figure 1:**
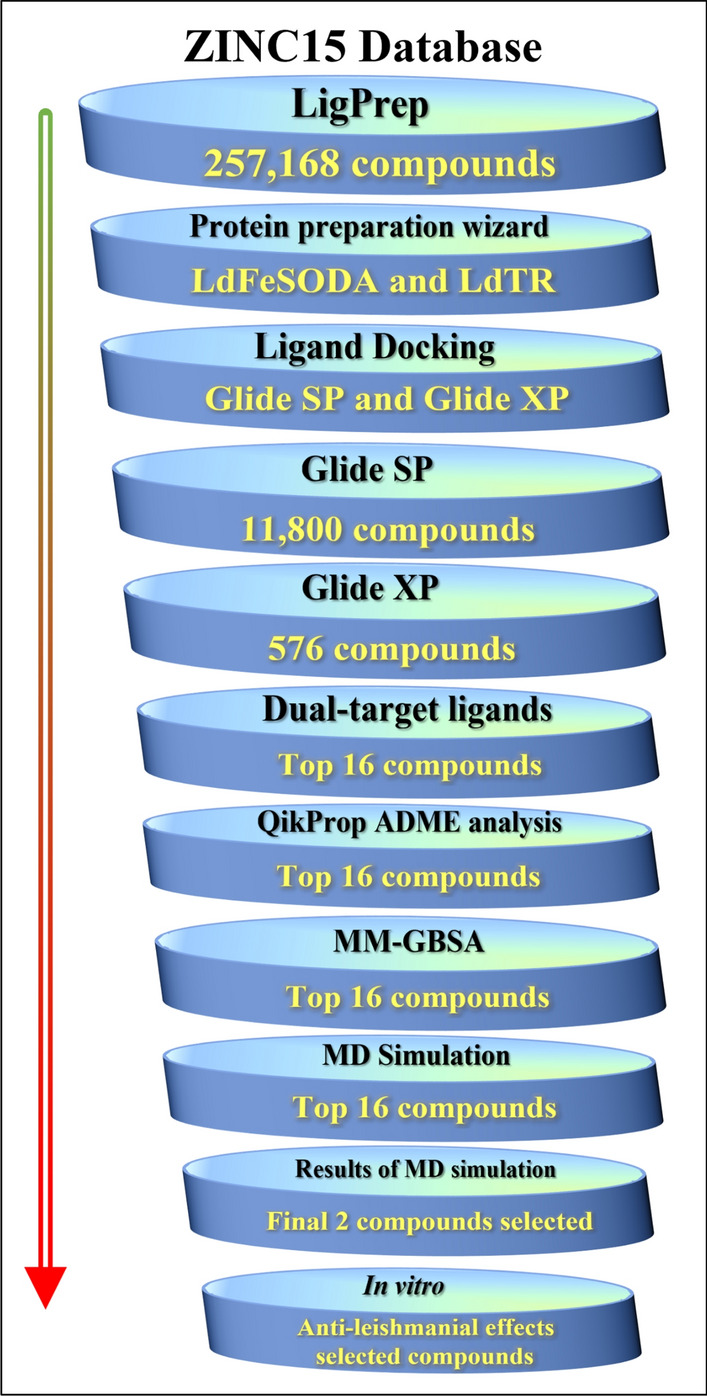
Schematic representation of the workflow for identification of dual-targeted inhibitor of LdFeSODA and LdTR.

This study explores the development of multitarget therapeutic molecules targeting two antioxidant defense enzymes, viz. FeSODA and TR, which neutralize ROS in parasites. By focusing on proteins crucial for parasite survival, yet absent in the human host, advancements in this field can be achieved. The Leishmania parasites contain trypanothione (TS_2_) [N1,N8-bis(glutathionyl)spermidine], a unique molecule involved in their redox metabolism. Because it acts as a major antioxidant and detoxifies harmful ROS produced by the host immune system, it is essential for parasite survival. Mammalian redox defense mechanisms are based on glutathione, while Leishmania parasites utilize trypanothione (TS) [N1,N8-bis(glutathionyl)spermidine]. TS_2_ is synthesized by trypanothione synthetase (TryS) and reduced by trypanothione reductase (TR). Inhibiting or disrupting trypanothione reductase activity can lead to toxic intermediate accumulation and oxidative stress, eventually causing parasite death^12^. Maintaining cellular redox potential relies on the crucial function of the enzyme superoxide dismutase (SOD, E.C.1.15.1.1). During parasite infection inside host macrophages, SOD plays a vital role in safeguarding the parasite against ROS^13^. The primary role of SOD is to convert superoxide radicals (O_2_^−^) into hydrogen peroxide (H_2_O_2_) and molecular oxygen (O_2_). Within the pathogens, iron superoxide dismutase (FeSOD) neutralizes the superoxide radicals (O_2_^−^) and prevents the creation of the peroxynitrite anion (ONOO^-^), enabling the pathogens to evade cytotoxic destruction reliant on redox reactions^14^. *L. donovani* possesses three distinct isoforms of SOD, viz FeSODA, FeSOD-B1 and FeSOD-B2, which play an important role in the dismutation of superoxide and converting in H_2_O_2_ and O_2_. The FeSODA isoform resides in the mitochondria, playing a role in cellular respiration. On the other hand, the isoforms FeSOD-B1 and FeSOD-B2 are found in the parasite's glycosomes, where they actively engage in metabolic pathways, including lipid biosynthesis, fatty acid oxidation, and glycolysis^15,16^. Maintaining the level of ROS is crucial in cellular respiration, and mitochondrial enzymes play a significant role in this process. Applying concept of multitarget drug discovery, our current study explores a single ligand molecule’s effectiveness against both trypanothione reductase and iron superoxide dismutase. This study utilized an advanced computational drug discovery method, which is considered one of the leading approaches in preclinical drug research. In particular, receptor-based virtual screening, which is also referred to as structure-based virtual screening, was utilized. The process involves comparing established protein structures with a database of ligands to identify potential candidates for pharmaceutical drugs as shown in Fig. 1. In order to identify the most favorable compounds, we conducted docking and post-docking screening of *L. donovani* LdFeSODA and LdTR, employing biogenic datasets. In addition to validating the inhibitory effect of selected compounds through virtual screening, we conducted an in vitro anti-leishmanial activity assessment using the MTT assay.

## Methodology

### Protein preparation

The protein structure of FeSODA and TR from *Leishmania donovani* is not available. However, these two proteins show very high similarity in other spices of the parasite. Accordingly, the crystal structures of FeSODA from *Leishmania major* (PDB ID: 4F2N) and TR from *Leishmania infantum* (PDB ID: 2JK6) were utilized in this study^17,18^. The protein preparation wizard, a feature of the Glide module Maestro 13.1, was used to prepare both protein structures. Preparation and refinement are the two parts of the protein preparation wizard. The protein preparation component first verifies chemical correctness, adds hydrogen, neutralizes side chains, and then assigns bond order using the Cambridge Structural Database (CSD). The protein preparation wizard was applied to form disulfide bonds, fill in missing atoms, improve side chains, and ensure the accuracy of partial atomic charges in order to produce the right structure. In the subsequent step, water molecules were removed, and a minimization process was executed until the average root mean square deviation (RMSD) of 0.3 Å for the non-hydrogen atoms was attained, employing the OPLS_2005 force field^19^.

### Ligand preparation

Biogenic compounds from the ZINC15 database (https://zinc15.docking.org/) were employed in this investigation as ligands for virtual screening. We utilized Maestro 13.1 to convert the Mol2 data for biological substances to Maestro format. All ligand molecules were generated using the LigPrep software, providing a diverse array of structures for each input structure, encompassing distinct ionization states, stereoisomeric properties, and tautomeric forms. It can also eliminate molecules based on the maximum ligand size of 500 atoms or keep certain chiralities for each input structure that has been processed. The optimization, which created the low-energy isomer of the ligands^20^.

### Molecular docking

The ligands were considered as flexible structures and the proteins as rigid structures during the docking experiments. For evaluating the docking parameters, we employed the Maestro 13.1 software’s Grid-based ligands docking with the Glide module. Following that, the active site of LdFeSODA (PDB ID: 4F2N) and LdTR (PDB ID: 2JK6) underwent docking with all ligands^21^. All docking studies were conducted without any constraints. Grid parameters were constructed and specified as − 2.89, − 21.01, and − 84.56 for LdFeSODA and 19.84, 42.96, and − 2.08 for LdTR as X, Y, and Z coordinates, respectively. The default grid size provided by the Glide programmer was utilized. The flexible glide docking method known as “Standard Precision” (Glide SP) was employed to perform the docking of the ligand with the active site^22^. Internally, Glide produces conformations that are then processed by a number of filters. The default hard potential function was used for the Glide docking settings. In SP docking, 50% of molecules were preserved in all conceivable states. To determine the optimal score condition, docking in extra precision (XP) mode with 100% retention was applied to each. To determine the flexibility of the ligands, Glide XP was employed to dock the ligands to the active sites of LdFeSODA and LdTR^23^. When the protein and ligand are accurately in hydrophobic contact, only the active small molecules will be able to escape the penalties and get favorable docking scores. The electrostatic energy interaction of the hydrogen bonds involved various elements, including side chains, backbone chains, salt bridges, and hydrophobic contacts. The visualization of 2-D and 3-D interactions between the selected compounds and the binding site residues was conducted using Maestro 13.1.

### Absorption, distribution, metabolism and excretion (ADME) analysis

QikProp was used to calculate the ADME (adsorption, distribution, metabolism, and excretion) characteristics and forecast the necessary principle and physiochemical descriptors of potential therapeutic molecules^24^. For the evaluation of ADME properties, as well as the potential drug activity according to the five rules of Lipinski's, the QikProp module was employed to analyze the pharmacokinetic spectacle of the five most significant biogenic compounds^25^.

### Molecular dynamics simulation

#### System building

Molecular dynamics (MD) simulations were conducted on the representative compounds of LdFeSODA and LdTR using the Desmond module of Maestro 13.1, after energy minimization^26^. To enhance protein interaction within the system, the OPLS_2005 force field was employed, and the TIP3P (transferable intermolecular potential with 3 points) water model was used to solvate the system^27^. To establish an appropriate setting, a water box with orthorhombic dimensions and a 20 Å buffer region was employed, ensuring a clear separation between the protein atoms and the box. Any overlapping water molecules were eliminated, and the system was neutralized through the addition of 0.15 M NaCl.

#### Molecular dynamics run

In order to investigate the dynamic movements and molecular behavior of specific ligand molecules in relation to LdFeSODA and LdTR, MD simulation studies were conducted. The protein–ligand systems were established by applying the OPLS_2005 force field parameters. The aim of the simulation was to establish a protein–ligand complex and assess its dynamic stability. Prior to commencing the MD simulation, a relaxation protocol consisting of five steps was employed, which included: (I) A Brownian dynamic simulation was performed using an NVT ensemble at a temperature of 310 K. The solute's heavy atoms were constrained using small timesteps, and the simulation was carried out for duration of 100 ps; (II) Following that, a second stage involved performing an NVT simulation at a temperature of 310 K. The solute’s heavy atoms were constrained using small timesteps, and the simulation was run for duration of 12 ps; (III) An NPT simulation at a temperature of 310 K was carried out, with a designated duration of 12 ps, during which restraints were applied to the heavy atoms of the solutes; (IV) An NPT simulation was executed for a duration of 12 ps, with restraints applied to the heavy atoms of the solutes; (V) An NPT simulation was carried out for a duration of 24 ps, without applying any restraints^28^. Throughout the dynamics phase, a multiple-time step RESPA integration algorithm was employed. The MD simulation was performed under the NPT ensemble, employing periodic boundary conditions to ensure a constant particle number, pressure, and temperature. In order to regulate the temperature, Nose–Hoover chain thermostat with a relaxation time of 1.0 ps was employed^29,30^. In order to ensure pressure stability throughout the simulation, the barostat system utilized a relaxation time of 2.0 ps with isotropic coupling, following the Martyna-Tuckerman-Klein method^31^. Throughout the entire simulation procedure, the temperature was maintained at a 310 K constant, at 1.0 bar pressure, and the pH at 7.0. To investigate the protein–ligand interactions, the simulation interaction diagram tool provided by the Desmond MD package was utilized. Throughout the simulation period, continuous monitoring of the RMSD (root mean square deviation) and RMSF (root mean square fluctuation) of the protein–ligand complex was conducted to evaluate any structural conformation changes.

### Binding free energy estimation

The binding energy of the LdFeSODA-ligands and LdTR-ligands complex was calculated using the MM-GBSA module of the Maestro 13.1 software. The MM-GBSA score was employed for this estimation. Throughout the procedure, the OPLS_2005 force field and the Variable-Dielectric Generalized Born Model (VSGB) solvation model were employed. The protein was maintained in a rigid state, whereas the ligand was treated as flexible in calculating binding free energy, denoted as ∆G _bind_^32,33^.

### Inhibition of *L. donovani* promastigotes growth

The strain of MHOM/IN/1983/AG83 *Leishmania donovani* culture was cultivated in M199 medium enriched with 10% FBS (fetal bovine serum) and penicillin 100 U/mL and streptomycin 100 µg/mL at a constant temperature of 25 °C. The culture was incubated for a period of 48 h with the objective of examining the impact of specific inhibitors on the growth of *L. donovani* promastigote cells, utilizing the 3-(4,5-dimethylthiazol-2-yl)2,5-diphenyltetrazolium bromide (MTT) assay. This assay is based on a yellow compound utilized, which undergoes reduction by mitochondrial enzymes in viable cells, resulting in the formation of a purple product called formazan. The test compounds, solubilized in dimethylsulfoxide (DMSO), were diluted in M199 medium and dispensed into 96-well plates. Each well was filled with a total volume of 200 µl. *L. donovani* promastigote cells (2 × 10^6^ cells/mL), which were previously cultured in M199 medium, were seeded into the wells containing different concentrations of the compounds. To serve as negative controls, cell cultures were kept in M199 medium without any administration of drugs. The negative control data was used for calculation % cell viability for each experiment. Positive controls were established using miltefosine concentrations that demonstrated complete inhibition. Following that, the cultures were transferred to a light-protected environment and incubated at a temperature of 25 °C for a duration of 48 h. After the treatment, the cultures were subjected to the addition of MTT reagent (0.5 mg/ml) and subsequently incubated in the absence of light at a temperature of 25 °C for a period of 4 h. After the completion of the incubation period, the plates underwent centrifugation at 4000 rpm for a duration of 45 min. The quantification of the resultant pellets was conducted by measuring the absorbance at a wavelength of 570 nm after dissolving the pellets, which contained formazan crystals, in DMSO. The determination of absorbance was performed using the BioTek Synergy HT microplate reader at a wavelength of 570 nm. To determine the IC_50_ values of each compound, a concentration–response curve was constructed by plotting the percentage of cell viability against the concentration of the compound. The resulting plot was analyzed to ascertain the IC_50_ values^34–37^. All experiments were repeated three times to ensure accuracy and reliability. For the IC_50_ analysis, a minimum of two independent experiments were conducted.

## Results

### Protein structure

For this study, the crystal structures of *Leishmania major* FeSODA (PDB ID: 4F2N) and *Leishmania infantum* TR (PDB ID: 2JK6) were obtained from the PDB database^13,14^. The crystal structure of 4F2N was resolved at a resolution of 1.85 Å with an R-value of 0.175. The active site of the structure encompassed residues His58, His108, Trp156, Asp191, Trp193, and His195. The crystal structure of 2JK6 has resolution 2.95 Å with an R-value of 0.237 and active side residues Cys52, Cys57, His461, Thr335. The 3D structure of crystal structure visualized with maestro 13.1.

### The compounds ZINC000008876351 and ZINC000253403245 show potential binding towards the targets

In this study, a total of 257,168 biogenic compounds were screened against LdFeSODA and LdTR using molecular docking method utilizing the Glide module integrated into the Schrodinger software. For virtual screening, Glide was employed with both the SP and XP configurations. Selection of ligands was based on their binding energy, with the objective of identifying ligands that exhibited similar affinities towards both LdFeSODA and LdTR. The drug demonstrates a binding affinity for both targets, which aligns with the fundamental principle of multitarget drug discovery. Two natural compounds were chosen based on their affinity to bind to both targets. The chosen compounds underwent computational analysis to assess their ADME properties and adherence to Lipinski's rule of five. The screening outcomes of natural compounds, considering factors such as molecular weight, Lipinski's rule violation, Log S, Log P, and SASA (Solvent Accessible Surface Area) are listed in Table 1. Further analysis was conducted using the commercially available compounds, specifically ZINC000008876351 and ZINC000253403245, which were selected for this purpose.

**Table 1:** Selected compounds complexed with LdFeSODA and LdTR underwent ADME analysis.

Compound name	Molecular weight (Da)	SASA	No. of H-bond donor	No. of H-bond acceptor	QPlogS	Log P	Lipinski violation
ZINC000008876351 (CID: 40,853,471)	514.534	784.344	4	8	− 2.728	1.611	1
ZINC000253403245 (CID: 110,206,972)	601.655	841.205	2	10	− 4.689	2.740	1

**Figure 2:**
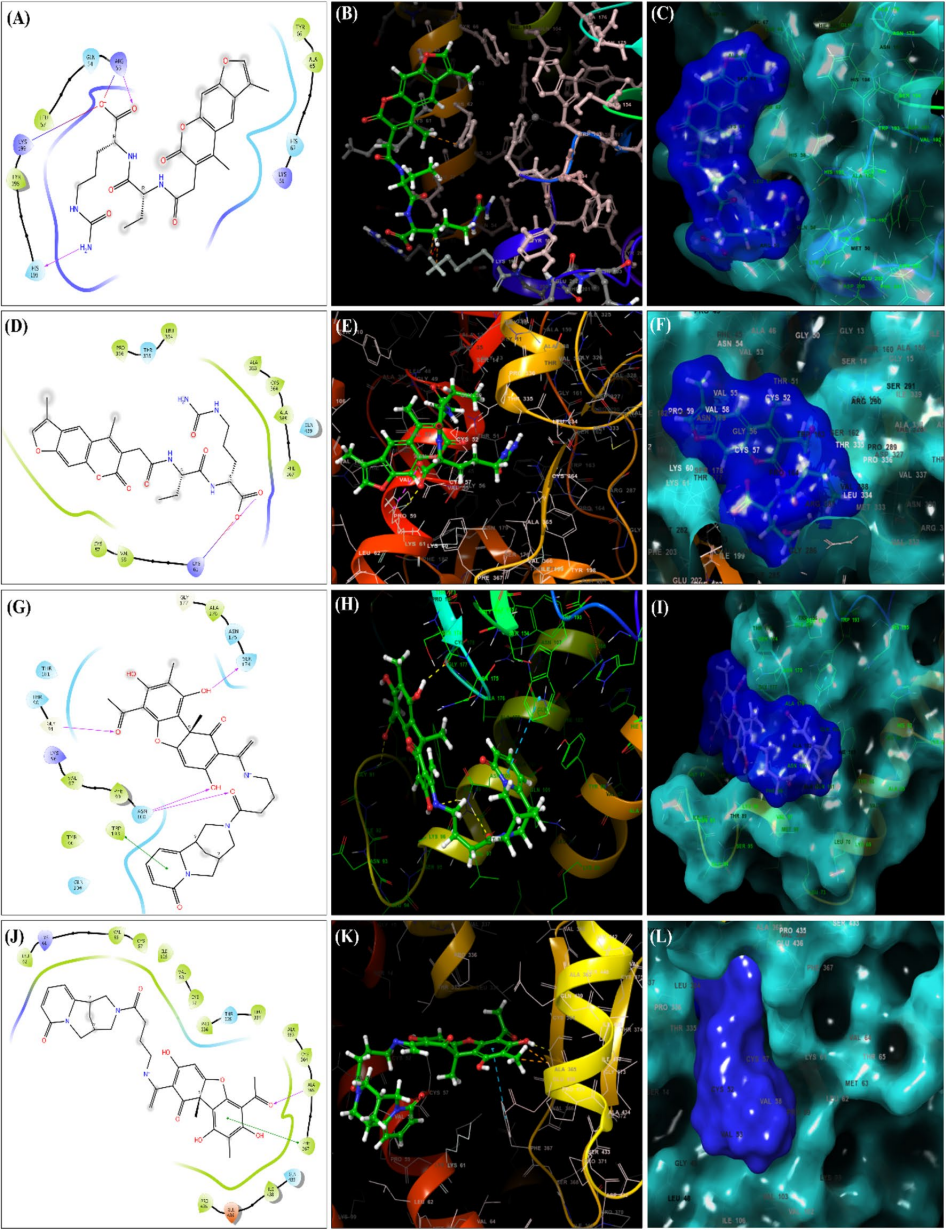
2-D and 3-D Interaction diagram of ligand–protein with Glide XP Docking. Interaction of the ligand ZINC000008876351 with *Leishmania donovani* FeSODA (**A**–**C**) and TR (**D**–**F**). Interaction of the ligand ZINC000253403245 with *Leishmania donovani* FeSODA (**G**–**I**) and TR (**J**–**L**).

The details of the docking score, glide emodel and glide gscore values obtained for ZINC000008876351 during its docking with the target protein 4F2N are provided in Table 2. The obtained scores were as follows for the docking of ZINC000008876351 with the target protein LdFeSODA (4F2N): docking score: − 6.994 kcal/mol, glide gscore: − 6.994 kcal/mol and glide emodel: − 51.145 kcal/mol. Similarly, when ZINC000008876351 targeted LdTR (2JK6), the corresponding scores were as follows: docking score: − 7.937 kcal/mol, glide gscore: − 7.937 kcal/mol and glide emodel: − 55.511 kcal/mol (Table 2). The results for the ligand ZINC000253403245 targeting LdFeSODA (4F2N) are as follows: docking score: − 5.834 kcal/mol, glide gscore: − 6.28 kcal/mol and glide emodel: − 63.554 kcal/mol (Table 2). Additionally, when ZINC000253403245 was docked with the protein LdTR (2JK6), the resulting scores were docking score: − 7.035 kcal/mol, glide gscore: − 7.035 kcal/mol and glide emodel: − 63.554 kcal/mol (Table 2). ZINC000008876351 exhibited interaction with the target protein LdFeSODA by forming two hydrogen bonds with the binding site residues Arg53 and His195 (Fig. 2A–C). Furthermore, it established a salt bridge between the binding site residues Arg53 and Lys199 (Fig. 2A–C). In the case of ZINC000008876351 docking with the targeted protein LdTryR (2JK6), it formed both a hydrogen bond and a salt bridge with the binding site residue Lys61 (Fig. 2D–F). ZINC000253403245 demonstrated interactions with the targeted protein LdFeSODA (4F2N) by forming four hydrogen bonds with the binding site residues Gly91, Ser174, and Asn100. Furthermore, it exhibited a π-π stacking interaction with the binding site residue Trp193 (Fig. 2G–I). In the case of ZINC000253403245 docking with the targeted protein LdTR (2JK6), it formed a hydrogen bond with the binding site residue Ala365 and a π-π stacking interaction with the binding site residue Phe367 (Fig. 2 J–L).

**Table 2:** Molecular docking analysis of selected ligand molecules against the target proteins LdFeSODA and LdTR.

Selected ligands	Targeted protein	Docking score (kcal/mol)	Glide gscore (kcal/mol)	Glide emodel (kcal/mol)
ZINC000008876351	FeSODA	− 6.994	− 6.994	− 51.145
	TR	− 7.937	− 7.937	− 55.511
ZINC000253403245	FeSODA	− 5.834	− 6.28	− 56.825
	TR	− 7.035	− 7.035	− 63.554

### Effects of selected compounds in human homologs hGR and hSOD2

Interactions of the specific compounds were also investigated with human homologs, namely glutathione reductase (hGR) and superoxide dismutase 2 (hSOD2), which are homologs to LdTR and LdFeSODA, respectively. Through a comparative docking analysis comparing LdFeSODA and hSOD2 interactions with the compound ZINC000008876351, which exhibited a weaker binding affinity for hSOD2, it was observed that compounds demonstrating stronger affinities for the designated target LdFeSODA were identified (Table 3). Similarly, for compound ZINC000253403245 with reduced affinity towards hSOD2, higher affinities for the chosen target LdFeSODA were evident (Table 3).

**Table 3:** Presents a comparative analysis of binding affinities between the target proteins LdFeSODA and LdTR, in contrast to their human homologs hSOD2 and hGR.

Selected ligands	Targeted protein	Binding affinity (kcal/mol)	Human homologs	Binding affinity (kcal/mol)	Difference in binding energy
ZINC000008876351	LdFeSODA	− 6.994	hSOD2	− 4.138	2.85
	LdTR	− 7.937	hGR	− 4.427	3.51
ZINC000253403245	LdFeSODA	− 5.834	hSOD2	− 3.855	1.97
	LdTR	− 7.035	hGR	− 3.723	3.31

Likewise, in the context of comparative docking analyses involving LdTR and hGR interactions with the compound ZINC000008876351, characterized by lower binding affinity for hGR, compounds displaying stronger affinities for the specific target LdTR were discerned (Table 3). Correspondingly, the compound ZINC000253403245, which exhibited weaker binding affinity for hGR, showcased compounds with increased affinities for the designated target LdTR (Table 3).

### Molecular dynamics analysis

In order to explore the protein–ligand complex conformational changes, MD simulations were performed for a duration of 100 ns on the complexes formed by ZINC000008876351 with the targeted protein LdFeSODA and ZINC000253403245 with the targeted protein LdTR, respectively. The trajectory obtained from the following complex MD run was analyzed for RMSD, RMSF, radius of gyration and H-bond interactions.

#### The protein shows stability during the MD simulation

**Figure 3:**
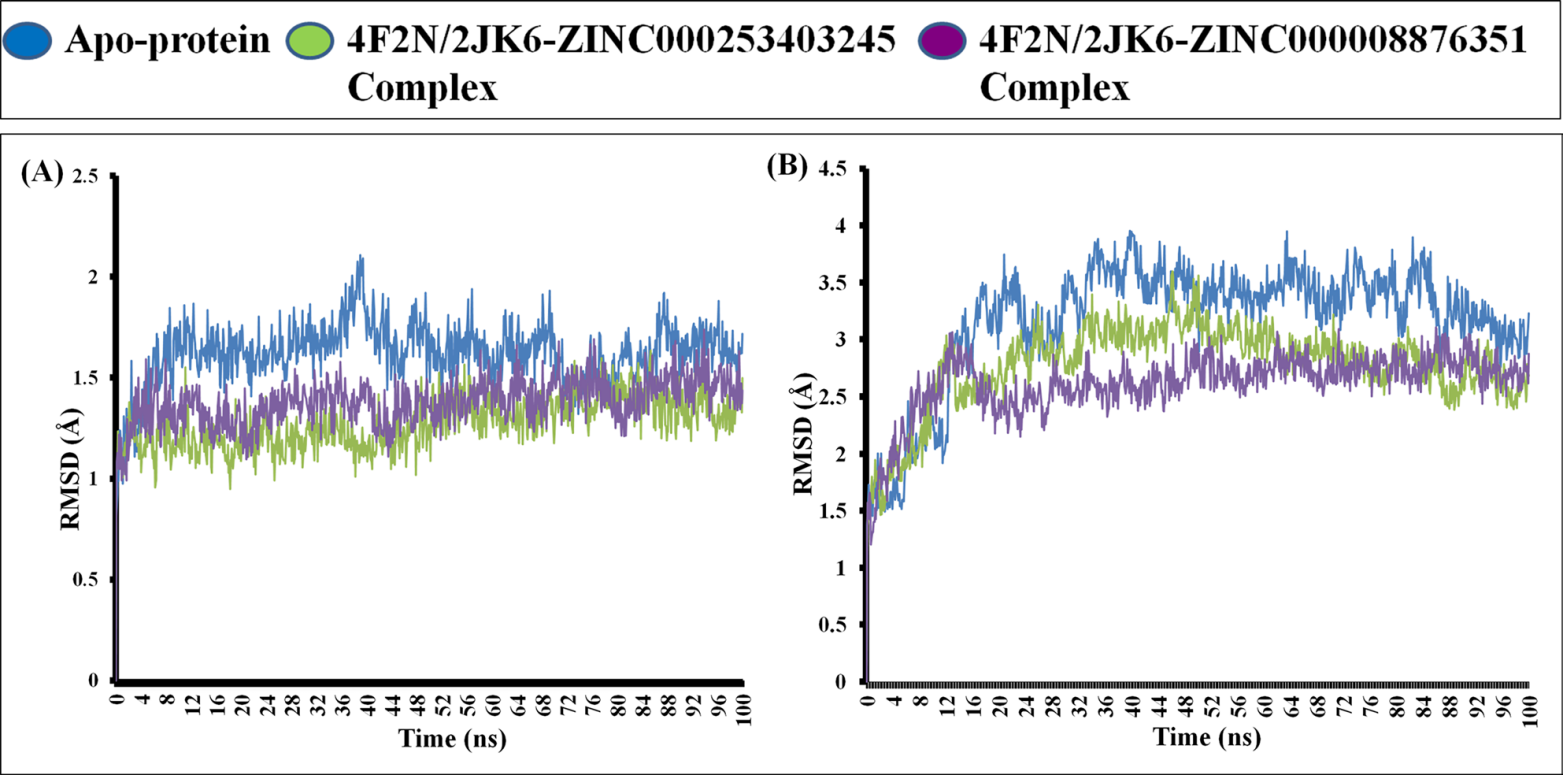
MD simulation for 100 ns comparative RMSD result analysis of apo-protein and protein–ligand complex with (**A**) analysis of the backbone RMSD trajectory; 4F2N apo-protein (Blue), 4F2N-ZINC000008876351 complex (Purple) and 4F2N-ZINC000253403245 (Green), respectively. (**B**) Analysis of the backbone RMSD trajectory; 2JK6 apo-protein (Blue), 2JK6-ZINC000008876351 complex (Purple) and 2JK6-ZINC000253403245 complex (Green), respectively.

During the entire 100 ns simulation run, the native protein of FeSODA exhibited stability with an average RMSD of 1.62 Å (Fig. 3A). Similarly, the native protein of TR displayed an average RMSD of 3.17 Å (Fig. 3B). Throughout the simulation run, the two selected ligands in the bound complexes demonstrated stability in the protein backbone. The ZINC000008876351-LdFeSODA complex exhibited an average RMSD of 1.38 Å, while the ZINC000008876351-TR complex showed an average RMSD of 2.615 Å (Fig. 3). Therefore, the protein in its ligand-bound forms exhibited lower deviations compared to the apo-protein. This indicates that the ligand–protein complex with smaller deviations is more stable. In the case of a small globular protein, a range of 1–3 Å for changes is considered acceptable. However, deviations exceeding this range indicate significant changes in the conformation of protein during the simulation. The ZINC000253403245-LdFeSODA complex exhibited an average RMSD of 1.276 Å, while the ZINC000253403245-TR complex showed an average RMSD of 2.768 Å (Fig. 3). During the course of the investigation, the RMSD values consistently exhibited stability, suggesting that the conformation of protein α-carbons remained relatively constant throughout the entire duration of the simulations. These findings strongly suggest that the ligands remained stable within the binding pocket of the target proteins and did not undergo significant diffusion or dissociation from the initial binding site.

#### The compound ZINC000008876351 exhibits higher stability

**Figure 4:**
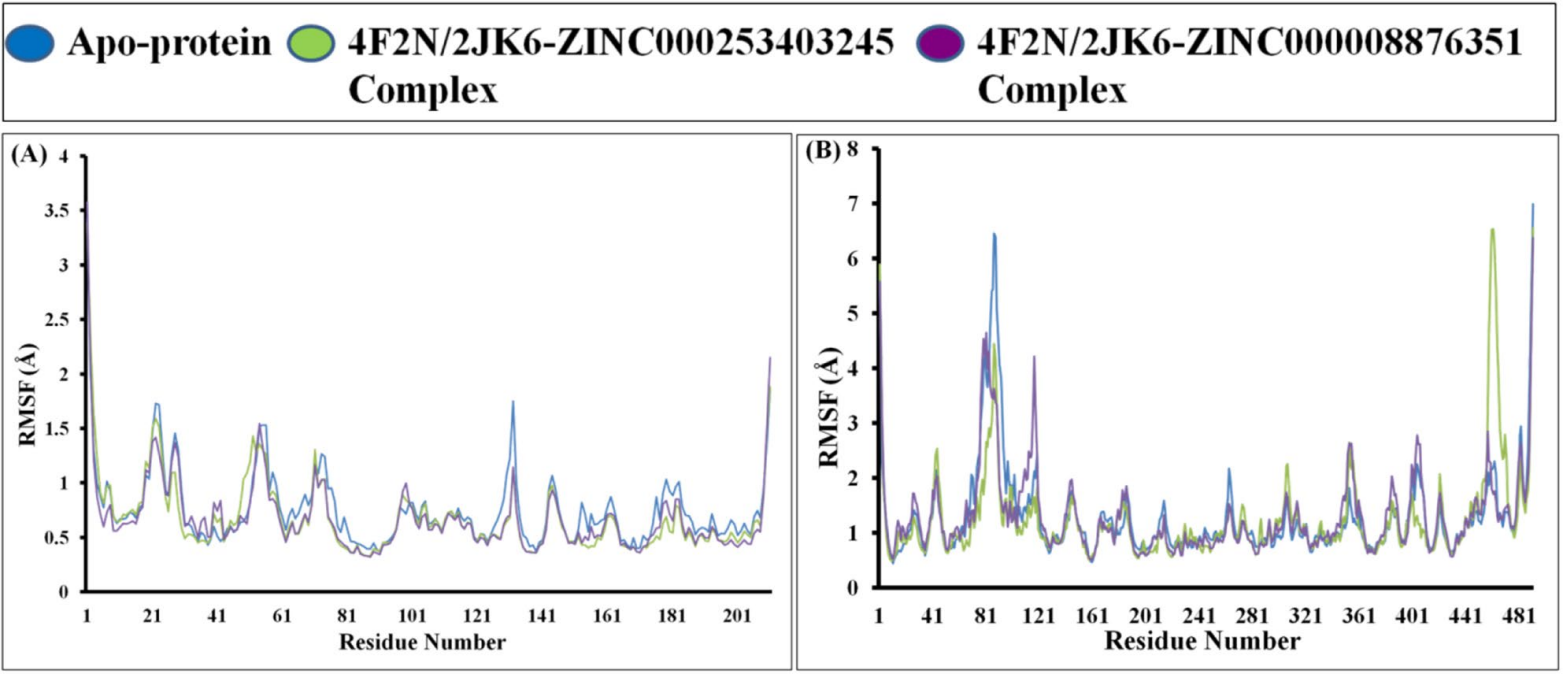
MD simulation for 100 ns comparative RMSF result analysis of apo-protein and protein–ligand complex with (**A**) Analysis of the backbone RMSF trajectory; 4F2N apo-protein (Blue), 4F2N-ZINC000008876351 complex (Purple) and 4F2N-ZINC000253403245 complex (Green), respectively. (**B**) Analysis of the backbone RMSF trajectory; 2JK6 apo-protein (Blue), 2JK6-ZINC000008876351 complex (Purple) and 2JK6-ZINC000253403245 complex (Green), respectively.

The Root Mean Square Fluctuation (RMSF) of the protein chain is employed as a metric to assess local conformational changes within the protein structure. The RMSF plot exhibited consistent secondary conformations that remained stable throughout the entire 100 ns MD runs. The average RMSF values were computed for the apo-protein 4F2N, the 4F2N-ZINC000008876351 complex, and the 4F2N-ZINC000253403245 complex, yielding values of 0.75, 0.67, and 0.68 Å, respectively (Fig. 4). The average RMSF values were determined for the apo-protein 2JK6, the 2JK6-ZINC000008876351 complex, and the 2JK6-ZINC000253403245 complex, resulting in values of 1.29, 1.30, and 1.25 Å, respectively (Fig. 4B). Distinct structural modifications can be observed in both the C-terminal and N-terminal regions of the protein. The active site residues of protein 4F2N (His58, His108, Trp156, Asp191, Trp193, and His195) and protein 2JK6 (Cys52, Cys57, His461, and Thr335) demonstrated notable stability, as indicated by their lower RMSF values (Fig. 4A). Within protein 2JK6, fluctuations detected in the region spanning residues 450–465 can be attributed to the presence of a beta loop, which is considered normal (Fig. 4B). The overall stability of the protein–ligand complex is indicated by the absence of significant conformational changes in the protein observed during the 100 ns molecular dynamics simulation.

#### Hydrogen-bond analysis

**Figure 5:**
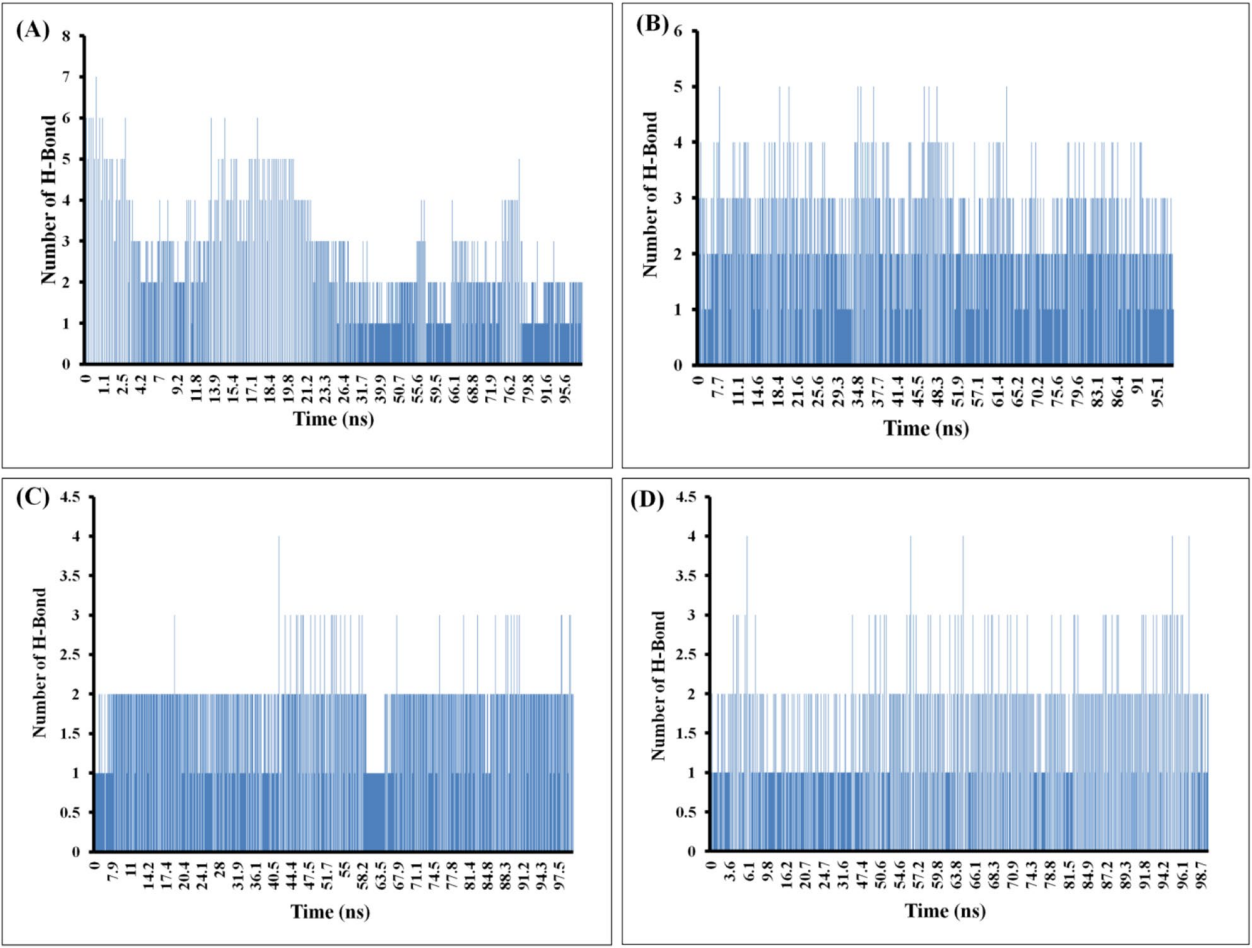
The following data presents protein–ligand complexes intermolecular hydrogen bonds counts throughout the entire 100 ns molecular dynamics simulation: (**A**) 4F2N-ZINC000008876351 complex, (**B**) 4F2N-ZINC000253403245 complex, (**C**) 2JK6-ZINC000008876351 complex, (**D**) 2JK6-ZINC000253403245 complex.

The protein–ligand complex is primarily characterized by hydrogen-bonding, which is a common and effective molecular interaction. To precisely ascertain the count of hydrogen bonds engaged in the interaction between the protein and ligand, a sequential procedure involving MD simulation followed by H-bond analysis was performed. The count of intermolecular H-bonds between the selected ligands (4F2N-ligands and 2JK6-ligands) during the MD simulation is shown in Fig. 5. This analysis provides insights into the overall stability of protein–ligand complex.

#### Radius of gyration (Rg) analysis

**Figure 6:**
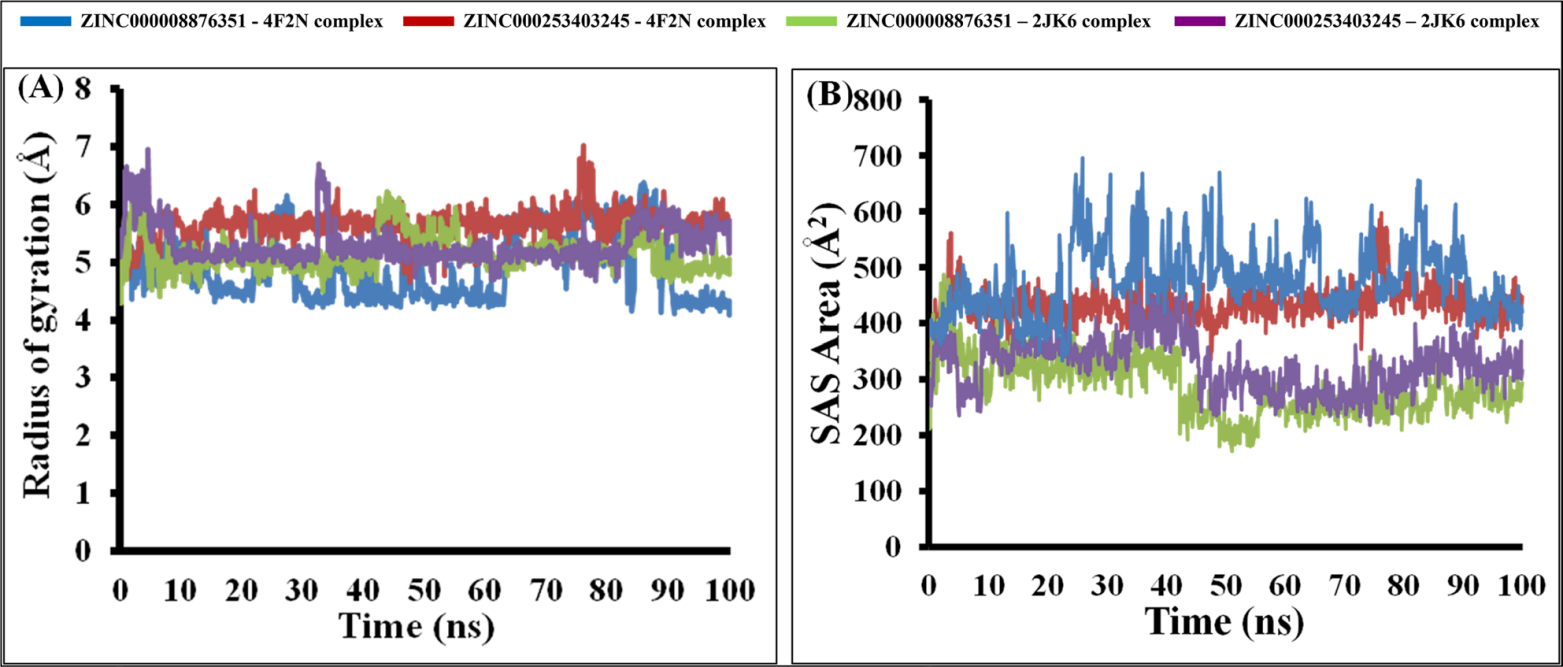
Graphical illustration of (**A**) Radius of Gyration and (**B**) SASA Analysis; ZINC000008876351-4F2N complex (Blue), ZINC000253403245-4F2N complex (Red), ZINC000008876351-2JK6 complex (Green), ZINC000253403245-2JK6 complex (Purple).

In order to assess the dimensions of the bound compounds, ZINC000008876351 and ZINC000253403245, in complex with the target proteins, their gyration radius (Rg) was computed. The gyration radius (Rg), which is indicative of the molecular size, the entire 100 ns MD simulation of each complex exhibited a constant value, suggesting that the size of the complexes remained stable over time. The average gyration radius (Rg) values were calculated for the complexes of ZINC000008876351 with 4F2N and 2JK6, resulting in values of 4.91 Å and 5.14 Å, respectively (Fig. 6A). Furthermore, the average Rg values for ZINC000253403245 in complex with 4F2N and 2JK6 were determined to be 5.65 Å and 5.31 Å, respectively (Fig. 6A). These results suggest that the ligands exhibit a similar shape and size in both the 4F2N and 2JK6 complexes, indicating the overall stability of the complex structures.

#### Solvent accessible surface analysis (SASA) analysis

By calculating the solvent accessible surface area (SASA) of protein–ligand complexes, it becomes possible to evaluate the surface area available for solvent interaction. This provides valuable insights into the interaction dynamics between the protein–ligand complex and solvent molecules. The average SASA score for the compound ZINC000008876351 in complex with 4F2N and 2JK6 was found to be 476.19 Å^2^ and 286.23 Å^2^, respectively (Fig. 6B). Likewise, the compound ZINC000253403245 in complex with 4F2N and 2JK6 displayed an average SASA of 433.69 Å^2^ and 325.67 Å^2^, respectively (Fig. 6B). Throughout the 100 ns MD simulation, a notable and consistent decrease in the SASA of all complexes was observed, signifying a state of structural uniformity (Fig. 5B). The chosen ligands, such as 2JK6, demonstrated SASA values below 350 Å^2^, whereas the selected ligands, including 4F2N, displayed SASA values above 400 Å^2^. The disparity could be ascribed to the greater size of LdTryR in comparison to LdFeSODA, enabling LdTR to interact with a larger number of solvent molecules.

#### Radial distribution function (RDF) analysis

**Figure 7:**
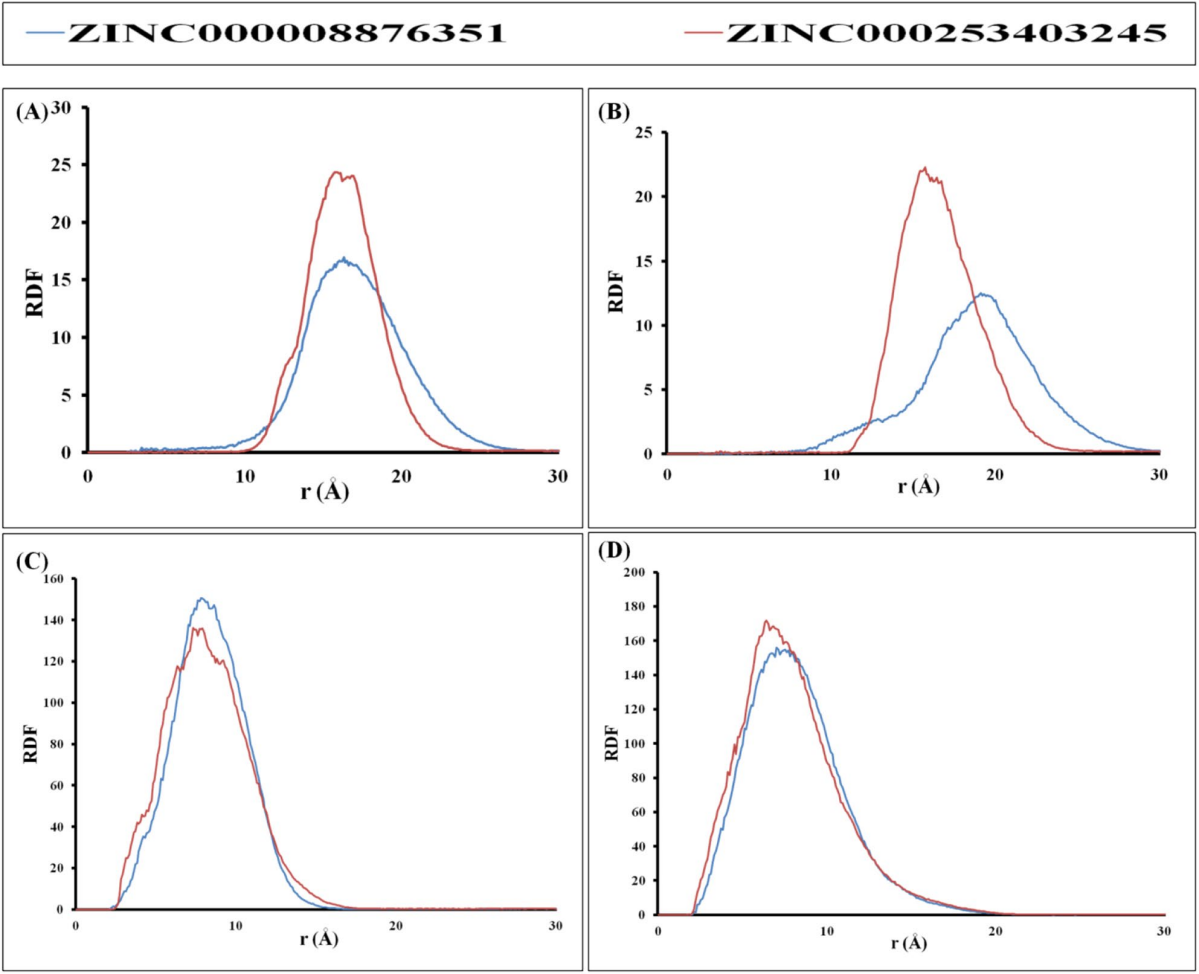
Graphical illustration of radial distribution function (RDF) Analysis; (**A** and **B**) LdFeSODA active site residue His108 and Asp191 RDF analysis with Ligand ZINC000008876351 (Blue), ZINC000253403245 (Red), respectively. (**C** and **D**) LdTR active site residue Cys52 and Thr335 RDF analysis with ligand ZINC000008876351 (Blue), ZINC000253403245 (Red), respectively.

In order to statistically measure the ligand's binding to the binding site and analyze the distribution of interaction radii that are crucial for maintaining the ligand's binding, the radial distribution function (RDF) is employed. To gain insights into the significance of ligand binding to the active site, RDF plots were generated for specific residues in LdFeSODA, namely His108 and Asp191, as well as for Cys52 and Thr335 in LdTR. The active site residue His108 in LdFeSODA exhibited 17.1 Å as the maximum distribution points with a 16.8 g(r) value for ZINC000008876351 and at 16.9 Å with a 24.02 g(r) value for ZINC000253403245 (Fig. 7A,B). The LdFeSODA active site residue Asp191 displayed 20.2 Å as the maximum distribution points with a 11.17 g(r) value for ZINC000008876351 and at 19.1 Å with a 12.49 g(r) value for ZINC000253403245 (Fig. 7A,B). Moreover, 7.5 Å is maximum distribution points with a 136.11 g(r) value for ZINC000008876351 and at 7.9 Å with a 150.40 g(r) value for ZINC000253403245 were observed for the LdTR active site residue Cys52. At 6.8 Å, ZINC000008876351 exhibited a 168.68 g(r) value for the active site residue Thr335 of LdTR, while at 7.5 Å, ZINC000253403245 showed a 154.81 g(r) value (Fig. 7C,D). The plots consistently demonstrate the involvement of active site residues in binding the stable compounds. The consistent patterns of interaction distances between the active site residues and the interacting ligands indicate that the binding modes of the compounds do not induce any alterations in the enzyme's active site.

#### Binding free energy estimation using ΔG_MM-GBSA_ method

The protein–ligand complexes binding free energy involving specific ligands and the targeted proteins LdFeSODA and LdTryR was estimated using the MM-GBSA method. An overview of the binding free energies of the protein–ligand complexes, including the individual components contributing to the binding free energy (ΔG_(MM-GBSA)_, ΔG_(ele)_, ΔG_(vdw)_, Δ_(GB)_, and ΔG_(non-polar)_), is provided in Table 4. Significantly, the ΔE (vdw) exerted a predominant influence on the stabilization of ligands within the protein binding site. Remarkable interactions were observed between the selected ligands and the targeted proteins in all four complexes, as evidenced by significant binding free energies.

**Table 4:** Free energy contribution protein-ligands complexes in MM-GBSA assay.

Protein–ligand complex	∆G_(MM-GBSA)_ (kcal/mol)	∆G_(vdw)_ (kcal/mol)	∆G _(ele)_ (kcal/mol)	∆G _(non-polar)_ (kcal/mol)	∆G _(GB)_ (kcal/mol)
ZINC000008876351-4F2N Complex	− 40.675	− 21.714	− 21.004	− 14.267	22.94
ZINC000008876351-2JK6 Complex	− 138.59	− 104.59	− 10.35	− 43.51	19.94
ZINC000253403245-4F2N Complex	− 56.223	− 39.241	− 17.147	− 24.503	9.028
ZINC000253403245-2JK6 Complex	− 141.34	− 105.15	− 4.674	− 44.32	4.82

#### Inhibition of *L. donovani* promastigotes growth

**Figure 8:**
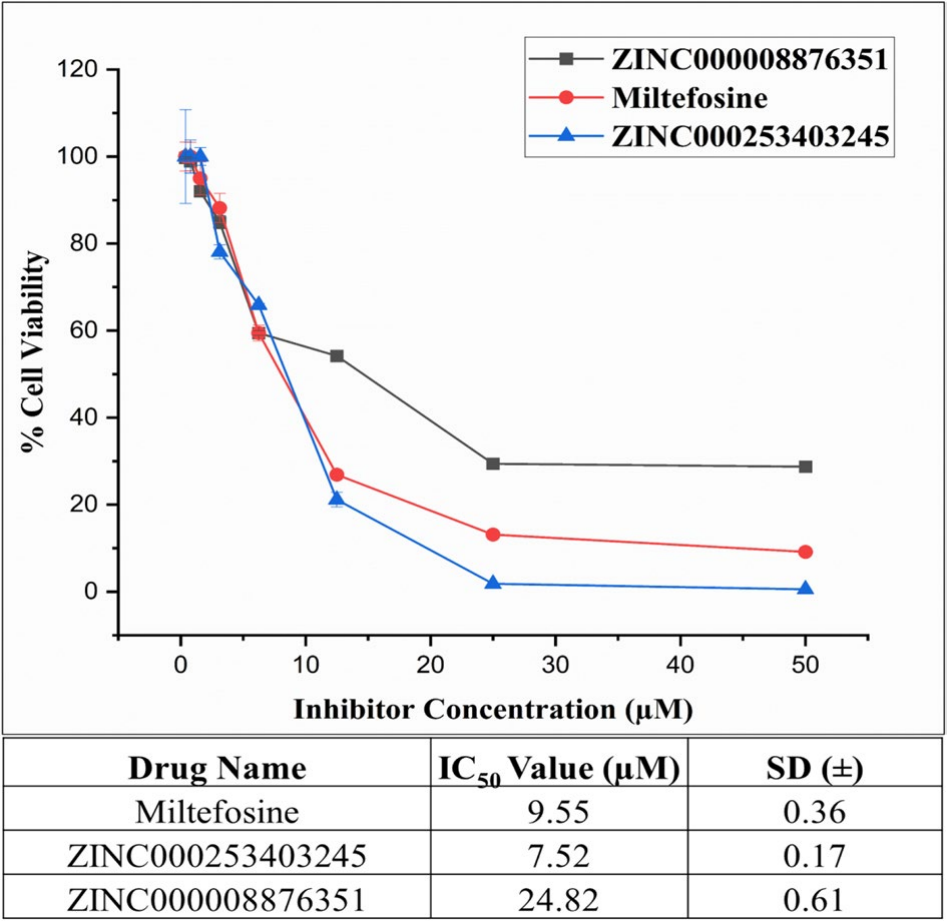
The Anti-leishmanial effects of selected Inhibitor on *L. donovani* promastigotes, *L. donovani* promastigotes treated with ZINC000008876351, ZINC000253403245 and Miltefosine dose range 0–50 µM for 48 h, all experiment perform in triplicate and IC_50_ was calculated by dose response curve in Origin software.

In a dose-dependent manner, both ZINC000008876351 and ZINC000253403245 exhibit effective inhibition of promastigote viability. To determine the IC_50_ values, a plot was created, correlating the concentration of the test compounds with the percentage of cell viability (Fig. 8). The measured IC_50_ value for ZINC000008876351 was 24.82 ± 0.61 µM, whereas for ZINC000253403245, it was determined to be 7.52 ± 0.17 µM. The commonly used drug Miltefosine the measure IC_50_ value was 9.55 ± 0.36 µM. For the negative control, a culture without drug treatment was used, while the concentration of Miltefosine that resulted in complete inhibition served as the positive control for promastigotes.

## Discussion

Visceral Leishmaniasis (VL) is a major worry for public health, especially in countries like India and Africa that are still developing. This is because factors like poverty, malnutrition, migration, and changes in the environment make people more likely to get the disease. For several years, the treatment options available for leishmaniasis have been constrained to a small number of selected chemotherapeutic agents, including miltefosine, antimonials, amphotericin, and paromomycin. Nevertheless, the current chemotherapy approaches have demonstrated inadequacy and dissatisfaction, emphasizing the necessity for a novel and more potent drug to combat leishmaniasis. Although drug combinations have shown initial effectiveness, their efficacy tends to diminish over time. Developing a new inhibitor for visceral leishmaniasis has proven challenging, prompting researchers to explore alternative treatment strategies. In recent literature reviews, diverse approaches have been identified, encompassing the utilization of immunomodulators, drug delivery systems based on nanotechnology, and the repurposing of drugs. Preclinical trials are currently underway to evaluate the efficacy of these strategies. Compared to traditional single-target drug discovery approaches, a multi-targeted drug discovery approach appears to be more promising. Multi-targeted molecules have the ability to interact with multiple targets simultaneously, by passing compensatory mechanisms and enhancing resilience against complex diseases.

Through the utilization of the ZINC database, we successfully identified two natural compounds that exhibit highly effective binding interactions with LdFeSODA and LdTR. In our endeavor to uncover novel anti-leishmanial compounds, we evaluated the comparative binding affinities of multiple biogenic compounds against the target Leishmanial proteins, specifically iron superoxide dismutase A (PDB ID: 4F2N) and trypanothione reductase (PDB ID: 2JK6). The process of molecular docking involves two successive methods, namely Standard Precision and Extra Precision, which are employed to determine the relative binding affinity of the chosen ligands. As a result of SP methods, about 11,800 ligands are retained, and these ligands are re-docked with target proteins as a result of XP methods. The extra precision (XP) docking method is more precise, and we obtained 16 ligands based on their docking score where we found an effective binding agent for the two targeted proteins, LdFeSODA and LdTR. Among these ligands, two demonstrated comparable affinities for the targeted proteins. Specifically, the ligand molecule ZINC000008876351 exhibited docking scores of − 6.994 kcal/mol and − 7.937 kcal/mol against proteins 4F2N and 2JK6, respectively. Similarly, another ligand molecule, ZINC000253403245, docked to proteins 4F2N and 2JK6 with docking values of − 5.834 kcal/mol and − 7.035 kcal/mol, respectively. The compounds under consideration underwent screening according to Lipinski's rule of drug likeness, aiming to ascertain their suitability for oral administration and minimal side effects. Among the five rules proposed by Lipinski, both compounds violate one rule by having a molecular weight exceeding 500 daltons. Nevertheless, straying from a single Lipinski's rule does not inherently eliminate the potential efficacy of a compound as a drug. This is particularly true due to the crucial role played by its target specificity and mechanism of action in combating Leishmania's antioxidant defense, which are pivotal factors in determining its effectiveness as a drug. Through the utilization of molecular dynamics simulation and predictions of relative binding affinity and binding energy against the target proteins, it was observed that the ligands ZINC000008876351 and ZINC000253403245 displayed the most notable binding affinities towards LdFeSODA and LdTR. Furthermore, the average RMSD values obtained for 4F2N and 2JK6 when bound to ZINC000008876351 were 1.38 Å and 2.615 Å, respectively. Similarly, the RMSD values obtained for 4F2N and 2JK6 when bound to ZINC000253403245 were 1.276 Å and 2.768 Å, respectively. These findings further confirm the strong binding interactions and stability of the ligand–protein complexes formed by ZINC000008876351 and ZINC000253403245 with LdFeSODA and LdTR. All the parameters from our simulation analysis show that the compounds are stable and efficient as multi-target compounds. By conducting a comparative analysis of binding affinities between the target proteins LdFeSODA and LdTR, in contrast to their human homologs hSOD2 and hGR, the potential toxicity of the selected compounds was predicted. While the targeted proteins were not present in the human host, human homologs hSOD2 showed ∼37% similarity, and hGR showed ∼35% similarity. The binding energy differences of the compounds suggested that both compounds exhibit varying binding affinity between parasite and human proteins, indicating potential selectivity. Concerns arise due to potential off-target effects on human proteins. To comprehensively assess the possible toxicity of these compounds, further studies are needed, including in vitro experiments to evaluate their effects on both parasite and human cells. In addition to that to validate above discussion, we perform in vitro anti-leishmanial activity by using MTT assay. The assay shows that to the increasing concentration of test compounds, viable cells number was decrease. Inhibitory concentration (IC_50_) values 24.82 ± 0.61 µM for ZINC000008876351 and 7.52 ± 0.17 µM for ZINC000253403245 was found, both the compounds effectively inhibit the growth of *L. donovani* promastigotes stage. The commonly used drug Miltefosine exhibited an IC_50_ value of 9.55 ± 0.36 µM, in comparison to the IC_50_ value found for selected inhibitor compound ZINC000253403245, which demonstrates a more potent inhibitory effect, indicating its potential as a strong therapeutic candidate. On the other hand, the compound ZINC000008876351 showed a less potent inhibitory effect than compound ZINC000253403245, but it still displayed moderate activity. We can conclude that the compounds ZINC000008876351 and ZINC000253403245 are effective inhibitors that can be utilized to treat visceral leishmaniasis.

## Conclusion

In this study, we have identified new multi-targeted anti-leishmanial compounds from the commercially available ZINC databases. These compounds have demonstrated binding capabilities with iron superoxide dismutase A (LdFeSODA) and trypanothione reductase (LdTR). The survival of parasites heavily relies on their antioxidant defense mechanisms as they encounter oxidative stress. Elevating reactive oxygen species (ROS) levels within parasite cells can render them more vulnerable to infection while also boosting the host's immune response. This oxidative burst in mitochondria or disruption of other cellular processes leads to parasite death. To identify potential compounds, we performed in silico screening of the Biogenic compounds database within the ZINC15 database. The evaluation of the compounds' ADME properties involved the utilization of QikProp, whereas the calculation of the relative binding free energy was conducted using MM-GBSA. The employment of molecular dynamics simulations allows for the investigation of the stability of protein–ligand complexes by studying the dynamic behavior of atoms or molecules under physiological conditions. In vitro experiments demonstrated the efficacy of the inhibitors against the growth of *L. donovani* promastigotes, with IC_50_ values of 24.82 ± 0.61 µM for ZINC000008876351 and 7.52 ± 0.17 µM for ZINC000253403245. The commonly used drug Miltefosine exhibited an IC_50_ value of 9.55 ± 0.36 µM, in comparison to the IC_50_ value found for selected inhibitor compound ZINC000253403245, which demonstrates a more potent inhibitory effect, indicating its potential as a strong therapeutic candidate. On the other hand, the compound ZINC000008876351 showed a less potent inhibitory effect than compound ZINC000253403245, but it still displayed moderate activity. Based on our findings, we can conclude that the natural compounds ZINC000008876351 and ZINC000253403245 are effective against our selected targets, LdFeSODA and LdTR. The identified compounds exhibit inhibitory effects on both enzymes, suggesting that compounds are good lead compounds to design new anti-leishmanial agents for the future treatment of visceral leishmaniasis.

## Data Availability

The datasets generated and/or analysed during the current study are already in the manuscript. Few raw data due to bulk size not uploaded are available from the corresponding author on reasonable request.
